# *Cardiocladius oliffi *(Diptera: Chironomidae) as a potential biological control agent against *Simulium squamosum *(Diptera: Simuliidae)

**DOI:** 10.1186/1756-3305-2-20

**Published:** 2009-04-24

**Authors:** Daniel A Boakye, Eric Fokam, Anita Ghansah, Josef Amakye, Michael D Wilson, Charles A Brown

**Affiliations:** 1 Noguchi Memorial Institute for Medical Research, Box LG581, Legon, Accra, Ghana; 2 Department of Life Sciences, University of Buea, Cameroon; 3 Water Research Institute, CSIR, P.O. Box 38, Achimota, Ghana

## Abstract

**Background:**

The control of onchocerciasis in the African region is currently based mainly on the mass drug administration of ivermectin. Whilst this has been found to limit morbidity, it does not stop transmission. In the absence of a macrofilaricide, there is a need for an integrated approach for disease management, which includes vector control. Vector control using chemical insecticides is expensive to apply, and therefore the use of other measures such as biological control agents is needed. Immature stages of *Simulium squamosum*, reared in the laboratory from egg masses collected from the field at Boti Falls and Huhunya (River Pawnpawn) in Ghana, were observed to be attacked and fed upon by larvae of the chironomid *Cardiocladius oliffi *Freeman, 1956 (Diptera: Chironomidae).

**Methods:**

*Cardiocladius oliffi *was successfully reared in the rearing system developed for *S. damnosum *s.l. and evaluated for its importance as a biological control agent in the laboratory.

**Results:**

Even at a ratio of one *C. oliffi *to five *S. squamosum*, they caused a significant decrease in the number of adult *S. squamosum *emerging from the systems (treatments). Predation was confirmed by the amplification of *Simulium *DNA from *C. oliffi *observed to have fed on *S. squamosum *pupae. The study also established that the chironomid flies could successfully complete their development on a fish food diet only.

**Conclusion:**

*Cardiocladius oliffi *has been demonstrated as potential biological control agent against *S. squamosum*.

## Background

Human onchocerciasis has been controlled as a disease of public health and socio-economic importance in parts of West Africa covered by the Onchocerciasis Control Programme (OCP), primarily through vector control. However, the blackfly vectors (various sibling species of *Simulium damnosum *Theobald complex) were not eliminated. Therefore, after cessation of vector control, re-colonization of the onchocerciasis-freed zones by the vectors has occurred, and this could lead to recrudescence of the disease if there are residual infections in the human population. These fears were allayed when mass treatment with ivermectin and the resultant massive reductions in microfilariae indicated that long-term treatments could lead to interruption of transmission. The return of the flies was therefore considered to pose only a biting nuisance and not a health risk, although anaphylactic shock, probably resulting in persons exposed to mass biting of two *Simulium *species in the English Midlands, has been reported [1].

There are no reports of the economic impact of the nuisance of *S. damnosum *s.l. in monetary terms [2]. However, Jamnback [3] reported that, in areas of major developmental projects, the incessant bites could be a serious threat to economic success. In Côte d'Ivoire, it was reported in the press (Jeune Afrique Economie, 1999, 106, 285) that farmers in the oncho-freed zones around the Leraba, Bou and Fombou rivers could only work between 1000 H and 1530 H due to *S. damnosum *s.l. biting nuisance. This translates into 2.5 hours of work lost, in addition to forcing the farmers to work during the hottest hours of the day. In Ghana, a possible negative impact of the biting nuisance on tourism was observed around the Kakum National Park during the rainy season in 1999 (Dr Kofi Ahmed et al, MOH unpublished report).

Recent information [4] indicates that elimination of transmission with ivermectin may not be feasible in all instances. There may be resistance to ivermectin in populations of *Onchocerca volvulus *[5]. Also, Boakye et al[6] showed that members of the *S. damnosum *s.l. can pick-up parasites at infection levels as low as 0.1–1.0 mf/mg skin and that these parasites could develop to the infective stage. Thus, apart from the biting nuisance, the presence of low levels of infection and the return of blackflies into the onchocerciasis freed zones could lead to a resurgence of onchocerciasis. There is therefore the necessity to continue to reduce blackfly density in areas of economic activity, as well as in the onchocerciasis-controlled areas of West Africa.

Large-scale vector control using chemicals is costly and, if not properly implemented, can result in serious environmental consequences. Hence, there is the need to find alternative methods that are cheap in the longer term, self-sustaining and relatively environmentally safe. The use of bio-control agents against pests of economic importance meets these criteria. Various organisms prey on, or infect, the different life stages of *S. damnosum *s.l., but few have been seriously evaluated for their usefulness as biological control agents. One group of organisms that need evaluating are larvae of the genus *Cardiocladius *(Family: Chironomidae), which are known predators of blackflies [7]. Unfortunately, none of the four species reported to breed in Africa has been recorded in West Africa [8], but recently *C. oliffi *was identified and described at an *S. damnosum *s.l. breeding site in Ghana and observed to feed on pupae of *S. damnosum *s.l. [9]. We report here the evaluation of this species in the laboratory as a potential biological control agent of *S. squamosum*.

## Methods

### Collection and identification of C. oliffi and S. squamosum

Egg masses of *C. oliffi *and *S. squamosum *were collected from Boti Falls (R. Pawnpawn (6°12'N and 0°13'W) in the rainy seasons (June – July) of 1999 and 2000. The samples were transported under moist conditions in wet plastic bags placed in an ice box to the laboratory for rearing to adults. Leaves with attached egg masses were cut into smaller pieces (approx. 2 cm^2^) and a thread passed through each piece and the thread attached to the tubing just above the air stone of the rearing system described in Boakye et al[10]. Rearing was done as described in Boakye et al[10]. Briefly, the tubing with the attached eggs was placed in a poly tube in a beaker filled with river water. The other end of the plastic tubing was fixed on to an Hyflo^® ^aeration pump. Air is pumped through the air-stone and the air bubbles coming through the poly tube creates a current sufficient to support the development of the aquatic stages of both the *S. squamosum *and *C. oliffi*. The rearing temperature was 28°C. The identification keys of Wiederholm [11,12] and Freeman [13] were used to identify *C. oliffi*. *Simulium squamosum *species were identified by cytotaxomy [14]. The chromosome slides for *S. squamosum *are deposited in the Parasitology Department of Noguchi Memorial Institute for Medical Research.

.

### Evaluation of C. oliffi as a biological control agent of S. squamosum

Five ratios of *C. oliffi *larvae to *S. squamosum *larvae; 0:25 (control), 1:5, 1:2, 1:1 and 25:0 were placed in different rearing systems and monitored for *S. squamosum *larval and pupal mortality and adult emergence. Each experiment started with 25 *S. squamosum *larvae per rearing system and the chironomid larvae varied with the exception of the system that had no *S. squamosum*. Six replicates were undertaken for each experimental regime except the last, which had four replicates. Fish food (Tetrafin^® ^Goldfish Flakes, Tetra, Blacksburg, VA) was provided daily in both the control and predation treatments. About 200 mg/tray of the feed was used in feeding the larvae. The rearing systems were cleaned everyday and the number of larvae and pupae dead or alive and adult emerged was recorded. The temperature during the trials was 28°C.

Significant differences between *S. squamosum *adults emerging from the separate treatment groups and the control were tested with one-way ANOVA using Dunnett's Method in SigmaStat 3.1 (Systat Software, USA).

### Demonstration of C. oliffi as a predator of S. squamosum

A positive implication of *C. oliffi *as a predator on *S. squamosum *was demonstrated using PCR-RFLP of a segment of the 28S ribosomal RNA gene. Genomic DNA was extracted from 1) eight larvae of *C. oliffi *not observed to have fed on *S. squamosum*, 2) thirteen larvae of *C. oliffi *observed to have fed on *S. squamosum *and 3) ten larvae of *S. squamosum *using the method of Flook et al[15]. PCR amplification of the gene segment of interest was done using the primers P1 (5'-TAGTGACGCGCATGAATGGA-3') and P2 (5'-GACGTCGCTATGAACGCTTGGC-3') [16] and following the above protocol [15]. The PCR products were visualised on 2% agarose gel stained in ethidium bromide.

Positive PCR products from the 3 experimental groups (unfed larvae of *C. oliffi*, fed larvae of *C. oliffi *and larvae of *S. squamosum*) were digested with the restriction enzyme *Alu*I following the manufacturer's instructions. The restriction fragments were visualised on 2% agarose gels stained with ethidium bromide, and the size variation recorded.

## Results

A total of 600 *S. squamosum *larvae and 358 *C. oliffi *larvae were used in the experiments. It was observed that larvae of *C. oliffi *attempted to grasp larvae of *S. squamosum *but were never successful as the *S. squamosum *larvae simply detached and moved away. However, these disturbances may have led to some larval mortality as indicated in the low numbers of pupae formed: 55 (36.6%), 42 (28%) and 28 (18.6%) in the experimental groups compared to the control group 96 (64%) (Table 1).

**Table 1:** Numbers and proportions of pupae and adults obtained for the different experimental groups.

Experimental group (*C. oliffi*:*S. squamosum*)	Number of larvae introduced in the rearing systems		Number of *S. squamosum *pupae (% of larvae)	Adults *S. squamosum *emerged		
			
	C. oliffi*	*S. squamosum*		Total number (Mean + SD)	% of larvae	% of Pupae
Control (0:25)	0	150	96 (64)	81 (14 ± 3.94)^a^	54.00	84.38
Treatment ii (1: 5)	30	150	55 (36.6)	22 (4 ± 2.94)^b^	14.67	40.00
Treatment iii (1: 2)	78	150	42 (28.0)	15 (3 ± 2.16)^b^	10.00	35.71
Treatment iv (1: 1)	150	150	28 (18.6)	1 (0.20 ± 0.48)^b^	0.67	3.57
Treatment v (25: 0)	100	0	-	-		

Total	358	600				

Values followed by different letters are significantly different (p < 0.05).

*There was no significant difference between the proportion of adult *C. oliffi *that emerged from the control and the proportion of adult of the same species emerging from the various treatments

The *C. oliffi *larvae were successful in feeding on pupae of *S. squamosum *because these were not mobile. A greater proportion (81 specimen, 54% of total) of adult *S. squamosum *flies emerged from the control compared with 22 (14.67%), 15 (10.0%) and 1 (0.67%) from the treatment groups. The difference was significant (ANOVA, *F *= 620, *p *< 0.0001, *df *= 3). Of the 358 *C. oliffi *larvae used in the experiments, 315 (87.99%) successfully completed their development to adults. There was no significant difference (*χ*^2 ^= 7.843, *df *= 6, *p *= 0.250) between the proportion of adult *C. oliffi *that emerged from the control and the proportion of adult of the same species emerging from the various treatments.

**Figure 1:**
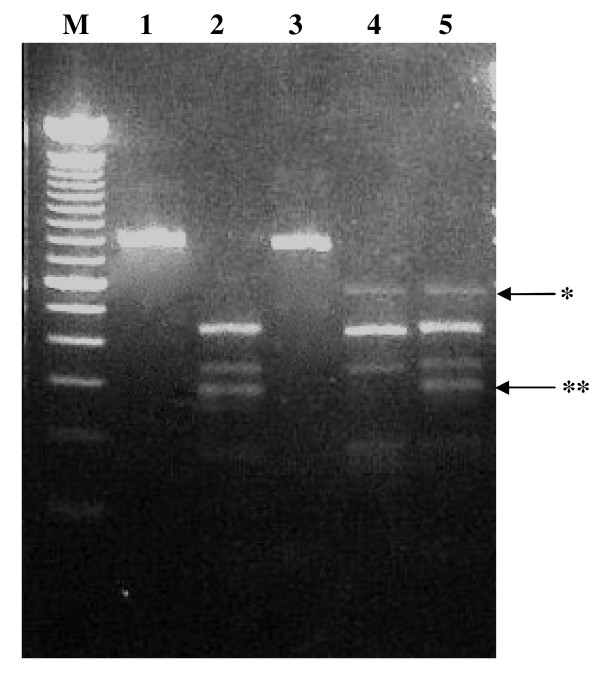
**P1/P2 PCR products and *Alu*I restriction digests**. *Alu*1 restriction digest of P1/P2 PCR products of *S. squamosum *and *C. oliffi *showing differences between the two species. Lanes: M = 100 bp ladder, 1 = Undigested P1/P2 *S. squamosum *PCR amplified product, 2 = *Alu *I digested P1/P2 *S. squamosum *PCR amplified product, 3 = Undigested P1/P2 *C. oliffi *PCR amplified product, 4 = *Alu *I digested P1/P2 *C. oliffi *PCR amplified product, 5 = *Alu *I digested P1/P2 *S. squamosum *fed *C. oliffi *PCR amplified product. * Band specific for C. oliffi. ** Band specific for *S. squamosum*.

The PCR generated fragments were approximately 800 bp for all the specimens, hence it was not possible to distinguish *S. squamosum *from *C. oliffi *upon electrophoresis (Fig 1). However, the *Alu*I restriction of the 800 bp PCR products showed variation in fragments sizes, indicating restriction site differences between the two species groups (Fig 1). It was therefore possible to show that *C. oliffi *had ingested *S. squamosum *by PCR amplification followed by *Alu*I digest (Fig 1 lane 5).

## Discussion

Benthic insects that prey on the *S. damnosum *complex have been identified in West Africa [17,18], and the Trichoptera of the family Hydropsychidae were considered important predators in Côte d'Ivoire. Similar observations were also made in Ghana [19]. However, none of these studies identified chironomids as predators of the *S. damnosum *complex leading Service and Elouard [17] to conclude that one Orthocladiinae species, that showed a positive precipitin reaction to *S. damnosum *antiserum, was probably due to contamination. This is therefore the first report of a chironomid preying on *S. damnosum *s.l. in West Africa. Outside of Africa, species of chironomids [20-23], particularly, larvae of *Cladiocladius *species [7], have been observed feeding on larvae and egg masses of black flies. The importance of predation of species of *Cladiocladius *on blackflies led to an attempt to use *C. australiensis *to control blackfly populations in Australia [24].

The use of molecular biological methods to implicate *C. oliffi *as a predator shows the potential of this technique for future identification of predators. The laboratory evaluation of *C. oliffi *as a potential biological control agent indicated that, whilst the predation by this species clearly resulted in fewer adult *S. squamosum *emerging from the systems, it could also complete its life cycle in the absence of *S. squamosum*. This means that *C. oliffi *is not an obligate predator on *S. squamosum*. Nevertheless, all of the *S. squamosum *egg masses collected from the study site were infested with *C. oliffi *during the collecting period. Although, predators may not provide effective control, they may nevertheless be important in regulating population size [17]. The predation of *C. oliffi *should therefore be assessed in a natural setting.

## Conclusion

This study has evaluated and demonstrated *C. Oliffi *as a potential biological control agent against *S. squamosum*. The study also established that the chironomid flies could successfully complete their development on a fish food diet only.

## Competing interests

The authors declare that they have no competing interests.

## Authors' contributions

All the authors have contributed substantially to this study. DAB and MDW contributed intellectually to the conceptualization, design and initiation of both the study and manuscript preparation. EF carried out the laboratory and field studies. CAB and AG advised and contributed to the laboratory studies and manuscript preparation. JA contributed towards the identification of larvae.
